# Supplementary material for: Developmental plasticity of Brachypodium distachyon in response to P deficiency: Modulation by inoculation with phosphate‐solubilizing bacteria
Source: Plant Direct. 2021 Jan 25;5(1):e00296. doi: 10.1002/pld3.296 (PMC7833465; doi:10.1002/pld3.296)
Supplement: Supplementary file 1 — Supplementary Material [file PLD3-5-e00296-s001.pdf]

Supplemental Resource 1 Biomass accumulation and RMF of four-week-old *Brachypodium* plantlets grown in Magenta boxes, exposed to contrasted P supplies and either inoculated or not inoculated with bacterial strains (n=30 for the P- and P+ treatments, n=15 for the P-/TCP and P-/HA treatments). Results of 3-way ANOVAs (degree of freedom "df", *P* and *F* values) and Dunnett's *post hoc* tests (annotated with stars; P+ and non-inoculated treatments used as references)

	Shoot biomass (mg)		Root biomass (mg)		Total biomass (mg)		RMF	
	mean	sd	mean	Sd	mean	sd	mean	sd
P treatment								
P-	78.65 *	17.53	57.78 *	13.43	136.43 *	27.98	0.42	0.05
P-/HA	92.09 *	29.20	51.25	11.08	143.34 *	35.52	0.37	0.06
P-/TCP	89.56 *	23.15	51.59	11.75	141.25 *	31.30	0.37	0.06
P+	138.25	33.14	51.00	12.81	188.90	41.99	0.27	0.04
ANOVA	df=3, *P*<2.2e-16, *F*=188.9276		df=3, *P*=4.278e-07, *F*=11.1453		df=3, *P*<2.2e-16, *F*=84.2410		df= 3, *P*<2.2e-16, *F*=313.3280	
Inoculation treatment								
Non-inoculated	112.05	36.78	55.02	14.04	167.07	41.82	0.34	0.08
AviF0819	108.42	39.08	56.00	13.06	164.42	44.44	0.35	0.08
BveFZB42	100.46 *	32.72	49.42 *	12.10	149.88 *	39.84	0.34	0.06
BveGB03	107.84	38.83	54.49	12.45	162.33	42.21	0.35	0.09
Eco99B829	103.56	34.44	54.28	11.58	156.97	35.83	0.36	0.08
Pfl29ARP	83.48 *	32.72	51.33	13.27	134.81 *	39.88	0.39	0.08
ANOVA	df=5, *P*=8.956e-16, *F*=17.2350		df=5, *P*=0.002956, *F*=3.6519		df=5, *P*=3.476e-11, *F*=12.2019		df=5, *P*=2.356e-15, *F*=16.7725	
Repetition								
ANOVA	df=1, *P*=0.2585, *F*=1.2795		df=1, *P*=0.013298, *F*=6.1721		df=1, *P*=0.1091, *F*=2.5765		df=1, *P*=0.2085544, *F*=1.5855	
Interaction								
P- non-inoculated	88.84	15.04	60.01	11.97	148.86	22.02	0.40	0.06
P- AviF0819	83.13	15.56	61.05	12.06	144.18	25.07	0.42	0.04
P- BveFZB42	80.21	19.12	51.28	13.53	131.49	31.51	0.39	0.03
P- BveGB03	79.81	13.45	61.35	10.44	141.16	21.41	0.43 *	0.04
P- Eco99B829	75.62	14.65	59.99	14.23	135.61	26.00	0.44 *	0.05
P- Pfl29ARP	64.34	17.60	53.11	15.08	117.44	30.64	0.45 *	0.05
P-/HA non-inoculated	100.09	25.94	55.54	13.70	155.62	31.15	0.36	0.08
P-/HA AviF0819	83.17	23.63	50.40	13.22	133.57	32.78	0.38	0.06
P-/HA BveFZB42	87.99	26.38	48.61	10.86	136.61	32.44	0.36	0.06
P-/HA BveGB03	101.44	35.68	49.55	9.45	150.99	40.80	0.34	0.07
P-/HA Eco99B829	101.27	32.89	54.15	8.14	155.43	37.93	0.36	0.06
P-/HA Pfl29ARP	78.59	24.21	49.23	9.98	127.83	32.08	0.40	0.06
P-/TCP non-inoculated	94.26	26.26	51.83	11.63	146.09	36.12	0.36	0.04
P-/TCP AviF0819	96.39	19.21	53.53	11.80	149.92	27.51	0.36	0.06
P-/TCP BveFZB42	93.72	20.98	48.94	11.43	142.65	29.81	0.34	0.04
P-/TCP BveGB03	90.40	24.71	53.77	14.86	144.16	37.03	0.37	0.04
P-/TCP Eco99B829	97.48	17.70	49.70	7.81	148.43	23.06	0.34	0.04
P-/TCP Pfl29ARP	65.7	14.42	51.70	12.92	117.40	23.81	0.44 *	0.05
P+ non-inoculated	150.13	31.20	51.36	16.15	201.49	43.82	0.25	0.05
P+ AviF0819	152.36	30.56	54.98	13.41	207.34	40.21	0.26	0.04
P+ BveFZB42	130.30	30.35	48.21	11.87	178.51	40.32	0.27	0.03
P+ BveGB03	147.60	30.07	50.53	11.90	198.13	38.98	0.25	0.04
P+ Eco99B829	135.48	28.99	50.50	9.14	183.69	32.12	0.28	0.04
P+ Pfl29ARP	113.97	32.82	50.40	13.33	164.36	42.14	0.31 *	0.05
ANOVA	df=15, *P*=0.1269, *F*=1.4330		df=15, *P*=0.735267, *F*=0.7482		df=15, *P*=0.6874, *F*=0.7917		df=15, *P*=0.0004245, *F*=2.7517	

Supplemental Resource 2 Coefficients, R^2 and *P* value of SMA lines (n=30 for the P- and P+ treatments, n=15 for the P-/HA and P-/TCP treatments), results of covariance analysis for differences among SMA lines coefficients (degree of freedom "df", *P* and likelihood ratio test "*LR*" values). If no significant difference was noticed between slopes, a common slope was used to test for difference in elevation. Treatments without any common letter are significantly different from each other (pairwise comparison)

	elevation	slope	R^2	*P*
Non-inoculated				
P-	0.51	0.81 *ab*	0.090	0.10762
P-/HA	-0.08	1.19 *a*	0.072	0.33277
P-/TCP	0.00	1.15 *a*	0.668	0.00019
P+	1.15	0.60 *b*	0.399	0.00018
Covariance analysis	/	df= 3, *P*=0.020606,		
P-				
Non-inoculated	0.38 *ab*	0.88	0.090	0.10762
AviF0819	0.34 *bc*		0.468	3.0804e-5
BveFZB42	0.39 *a*		0.718	3.5339e-9
BveGB03	0.32 *cd*		0.460	5.3114e-5
Eco99B829	0.31 *cd*		0.411	0.00013
Pfl29ARP	0.28 *d*		0.604	4.3258e-7
Covariance analysis	df=5, *P*=2.4697e-9, *LR*=48.77	df=5, *P*=0.75661, *LR*=2.631		
P-/HA				
Non-inoculated	-0.71	1.56	0.072	0.33277
AviF0819	-0.73		0.394	0.01228
BveFZB42	-0.69		0.264	0.05028
BveGB03	-0.65		0.227	0.07242
Eco99B829	-0.71		0.384	0.01371
Pfl29ARP	-0.75		0.582	0.00093
Covariance analysis	df=5, *P*=0.43264, *LR*=4.865	df=5, *P*=0.17355, *LR*=7.7		
P-/TCP				
Non-inoculated	0.23 *ab*	1.02	0.668	0.00019
AviF0819	0.23 *ab*		0.249	0.05839
BveFZB42	0.25 *ab*		0.513	0.00269
BveGB03	0.19 *b*		0.630	0.00069
Eco99B829	0.27 *a*		0.339	0.03689
Pfl29ARP	0.08 *c*		0.419	0.00905
Covariance analysis	df=5, *P*=4.4342e-7, *LR*=37.65	df=5, *P*=0.83515, *LR*=2.1		
P+				
Non-inoculated	1.15	0.60 *c*	0.399	0.00018
AviF0819	0.93	0.72 *bc*	0.312	0.00132
BveFZB42	0.45	0.99 *ab*	0.621	2.3175e-7
BveGB03	0.79	0.81 *bc*	0.512	1.2825e-5
Eco99B829	0.17	1.15 *a*	0.312	0.00165
Pfl29ARP	0.10	1.15 *a*	0.529	5.3439e-6
Covariance analysis	/	df=5, *P*=0.00842, *LR*=15.5		

Supplemental Resource 3 TRL of four-week-old *Brachypodium* plantlets grown in Magenta boxes, exposed to contrasted P supplies and either inoculated or not inoculated with bacterial strains (n=18 for the P- and P+ treatments, n=9 for the P-/HA and P-/TCP treatments). Results of 3-way ANOVAs (degree of freedom “df”, *P* and *F* values) and Dunnett’s *post hoc* tests (annotated with stars; P+ and non-inoculated treatments used as references)

	TRL (cm)	
	mean	sd
P treatment		
P-	224.58	42.78
P-/HA	233.06*	42.37
P-/TCP	213.26	49.83
P+	213.87	46.39
ANOVA	df=3, *P*=0.02126, *F*=3.2821	
Inoculation treatment		
Non-inoculated	237.85	48.83
AviF0819	228.11	42.30
BveFZB42	214.92*	50.62
BveGB03	233.41	40.92
Eco99B829	213.11*	35.64
Pfl29ARP	195.82*	41.59
ANOVA	df=5, *P*=1.99e-06, *F*=7.2513	
Repetition		
ANOVA	df=1, *P*=0.07758, *F*=3.1363	
Interaction		
P- non-inoculated	252.83	40.23
P- AviF0819	239.12	33.89
P- BveFZB42	214.76	49.38
P- BveGB03	234.62	31.18
P- Eco99B829	218.86	26.65
P- Pfl29ARP	187.32	42.97
P-/HA non-inoculated	257.96	31.15
P-/HA AviF0819	219.12	39.79
P-/HA BveFZB42	231.37	40.44
P-/HA BveGB03	235.36	49.72
P-/HA Eco99B829	248.26	45.64
P-/HA Pfl29ARP	206.27	33.97
P-/TCP non-inoculated	198.82	76.65
P-/TCP AviF0819	221.07	38.39
P-/TCP BveFZB42	224.41	61.96
P-/TCP BveGB03	222.32	51.99
P-/TCP Eco99B829	207.66	28.38
P-/TCP Pfl29ARP	205.30	33.29
P+ non-inoculated	232.32	36.08
P+ AviF0819	225.11	52.78
P+ BveFZB42	202.13	51.09
P+ BveGB03	236.76	41.63
P+ Eco99B829	192.52	27.13
P+ Pfl29ARP	194.36	47.95
ANOVA	df=15, *P*=0.15092, *F*=1.3891	

Supplemental Resource 4 Results of PERMANOVA performed on the persistent homology analysis output of plant root systems. n=18 for the P- and P+ treatments, n=9 for the P-/HA and P-/TCP treatments. Post-hoc tests were performed by running a PERMANOVA for each pairwise comparison and *P* values were adjusted for multiple comparisons using the Benferroni method.

	Df	*F* model	*P*
Inoculation treatment	2	11.1650	0.000999
P treatment	3	2.8237	0.005994
Interaction	6	1.2461	0.217782
Residuals	150		

Post-hoc tests:

	F model	*P*
P- vs P-/TCP	1.8911	0.68931
P- vs P+	2.1396	0.60539
P- vs P-/HA	2.5906	0.28771
P-/TCP vs P+	3.1768	0.19780
P-/TCP vs P-/HA	1.4693	1.00000
P+ vs P-/HA	3.5882	0.17982
Pfl29ARP vs non-inoculated	18.7287	0.00099
Pfl29ARP vs BveFZB42	13.9209	0.00099
Non-inoculated vs BveFZB42	1.0035	0.38462

Supplemental Resource 5 Shoot P concentration and PUE parameters of four-week-old *Brachypodium* plantlets grown in Magenta boxes, exposed to contrasted P supplies and either inoculated or not inoculated with bacterial strains (n=6 for the P- and P+ treatments, n=3 for the P-/HA and P-/TCP treatments). Results of 3-way ANOVAs (degree of freedom “df”, *P* and *F* values) and Dunnett’s *post hoc* tests (annotated with stars; P+ and non-inoculated treatments used as references)

	Shoot P concentration (µg P/mg FW)		PUpE		PUtE		PPUE	
	mean	sd	mean	sd	Mean	Sd	mean	sd
P treatment								
P-	0.237*	0.032	2418.692*	662.318	4.296*	0.603	1664.549*	294.805
P-/HA	0.335*	0.064	4146.142*	1768.202	3.099*	0.626	1365.267*	135.433
P-/TCP	0.281*	0.092	3318.446*	1565.915	3.940*	1.267	1649.241*	361.100
P+	0.764	0.086	337.427	60.022	1.325	0.151	915.617	222.696
ANOVA	df=3, *P*<2e-16, *F*=292.0433		df=3, *P*<2e-16, *F*=306.9495		df=3, *P*<2e-16, *F*=217.6723		df=3, *P*<2.2e-16, *F*=60.9819	
Inoculation treatment								
Non-inoculated	0.432	0.233	2444.236	2012.003	3.026	1.488	1501.401	467.108
AviF0819	0.421	0.243	2110.551	1542.716	3.136	1.493	1482.431	419.206
BveFZB42	0.457	0.260	2255.018	1662.922	2.886	1.410	1304.184	436.268
BveGB03	0.440	0.248	2247.915	1942.801	2.997	1.443	1354.915	360.533
Eco99B829	0.457	0.261	2350.842	2048.284	2.937	1.485	1329.075	453.353
Pfl29ARP	0.412	0.256	1568.265*	1258.735	3.298	1.543	1202.833*	381.678
ANOVA	df=5, *P*=0.2825, *F*=1.2751		df=5, *P*=0.01069, *F*=3.2079		df=5, *P*=0.4357, *F*=0.9788		df=5, *P*=0.007738, *F*=3.3921	
Repetition								
ANOVA	df=1, *P*=0.6384, *F*=0.2225		df=1, *P*=0.83696, *F*=0.0426		df=1, *P*=0.4312, *F*=0.6257		df=1, *P*=0.648586, *F*=0.2092	
Interaction								
P- non-inoculated	0.234	0.031	2704.936	682.528	4.350	0.608	1913.742	245.850
P- AviF0819	0.228	0.037	2476.670	739.706	4.487	0.686	1841.606	221.698
P- BveFZB42	0.255	0.037	2664.388	746.187	4.009	0.710	1588.721	289.616
P- BveGB03	0.239	0.040	2412.522	692.725	4.284	0.701	1631.159	186.833
P- Eco99B829	0.232	0.033	2275.638	540.169	4.378	0.607	1649.714	296.266
P- Pfl29ARP	0.236	0.022	1977.999	544.750	4.268	0.430	1362.351	248.987
P-/HA non-inoculated	0.357	0.079	4775.731	2043.682	2.896	0.664	1395.929	126.322
P-/HA AviF0819	0.305	0.044	3327.955	1067.802	3.323	0.456	1361.300	58.025
P-/HA BveFZB42	0.344	0.046	3942.647	989.521	2.944	0.416	1283.964	129.002
P-/HA BveGB03	0.363	0.076	4969.660	2542.434	2.836	0.608	1374.750	154.550
P-/HA Eco99B829	0.343	0.069	4684.583	2454.629	2.998	0.602	1449.523	212.164
P-/HA Pfl29ARP	0.295	0.089	3176.275	1807.334	3.594	1.055	1326.138	163.137
P-/TCP non-inoculated	0.296	0.117	3770.126	2229.912	3.826	1.715	1722.268	566.133
P-/TCP AviF0819	0.285	0.089	3654.221	1536.643	3.792	1.388	1765.498	368.753
P-/TCP BveFZB42	0.286	0.095	3586.993	1625.492	3.835	1.530	1714.052	354.350
P-/TCP BveGB03	0.266	0.067	2989.873	1264.261	3.944	1.112	1606.530	157.781
P-/TCP Eco99B829	0.351	0.141	4186.080	1884.872	3.296	1.666	1460.423	611.666
P-/TCP Pfl29ARP	0.203	0.020	1723.384	157.770	4.947	0.453	1626.672	188.967
P+ non-inoculated	0.736	0.062	354.844	41.169	1.368	0.114	1031.362	204.255
P+ AviF0819	0.741	0.088	363.894	57.738	1.365	0.163	1042.288	220.108
P+ BveFZB42	0.802	0.086	335.845	48.824	1.260	0.138	824.824	180.071
P+ BveGB03	0.765	0.078	351.455	70.629	1.318	0.129	942.947	232.530
P+ Eco99B829	0.791	0.111	341.557	38.802	1.286	0.186	882.537	248.321
P+ Pfl29ARP	0.751	0.102	276.967	73.901	1.354	0.193	769.744	189.371
ANOVA	df=15, *P*=0.5820, *F*=0.8858		df=15, *P*=0.72824, *F*=0.7489		df=15, *P*=0.9355, *F*=0.4976		df=15, *P*=0.937471, *F*=0.4938	
