# Supplementary material for: Developmental plasticity of Brachypodium distachyon in response to P deficiency: Modulation by inoculation with phosphate‐solubilizing bacteria
Source: Plant Direct. 2021 Jan 25;5(1):e00296. doi: 10.1002/pld3.296 (PMC7833465; doi:10.1002/pld3.296)
Supplement: Supplementary file 2 — Supplementary Material [file PLD3-5-e00296-s002.pdf]

Reviewer #1:

I think the experiments are sound, however the conclusions are too strong given the data. Text could be simplified to give the reader a clear idea of how PSB changes Brachypodium development.

Thank you for your comment. We checked our conclusions and revised the text of the manuscript to make sure that our conclusions are supported by the data and provide additional information about how PSB change *Brachypodium* development.

Reviewer #2:

Overall, this study is an important contribution using a variety of methods to understand whole plant growth, including shoots and roots, in the context of soil fertility and microbial interactions. I found the study and interpretations to be mostly sound, but do request some details and clarifications below.

Thank you for your detailed comments. The requested clarifications are given below and the manuscript was adapted where necessary.

This manuscript would benefit from providing a bit more technical background for diverse readership. Many terms used like gnotobiotic and axenic may not be known by, at least, plant scientists who don't work with microbes. Discussing these in the introduction and why it's important would be warranted. Adding some details about solubility processes involving the various P forms and their mechanisms would also be good, including processes in natural soil like fixation and how microbes solubilize. In the methods, I think a sort of introductory paragraph explaining the overall goal and methods would be helpful that also defines the commonly used terms within the paper like TCP and HA.

We agree that such an introductory paragraph would be useful to the reader. The section "2.1 Objectives and methodology" was added. This section gives information about the P forms TCP and HA, the co-cultivation system, and the choice of the methods used to analyse root biomass allocation and root system morphology. The part describing allometric analyses was moved from the section "statistical analyses" to avoid repetition.

The novelty of using persistent homology as an additional metric is appreciated, but a bit more description of what the metric means is needed and why it's more helpful than say only measuring root length, biomass, or something like the branching or topology?

Root system morphology can be described in different ways. So far, root systems have mainly been characterised using geometric descriptors such as total root length or number of root tips for instance. However, such descriptors often fail to describe the full complexity of root systems. The main reason for this is that another component of root system morphology, which is topology (i.e., the way individual roots are connected to each other through branching), is often not considered. Two root systems can look very similar from a geometric perspective (same total root length and same number of root tips) but have very different topologies.

Persistent homology is a mathematical method allowing the quantification of plant morphology. The idea behind this method is to create a barcode encapsulating geometric and topological information for each root system (see interactive Figure 3 in Delory et al. 2018). For instance, the number of horizontal bars in a barcode is equal to the number of root tips, and the sum of the length of all horizontal bars in a barcode is equal to the total root

system length. In addition, topological information is encapsulated by the birth/death of each branch along a geodesic distance function.

We argue that an analysis of plant root systems using persistent homology nicely complements more classic approaches relying on geometric descriptors.

Seems likes a useful study system. Nice job.

Thank you.

25: P is not defined.

It is now defined.

28: I had to look up gnotobiotic, so would be good to either define or just use simpler terms to describe the system.

The term is replaced by “*in vitro*” in the abstract to avoid a long definition. The term is now defined in the introductory paragraph of the Material & Methods section.

32: Not a big deal, but I think allocation pattern is a bit redundant - pattern is not needed

We do not consider these two words redundant. The term “allocation” is used to name the repartition of the biomass between organs. “Pattern” refers to the used scheme to allocate the resources.

~~82~~83: I think it'd be good to define 'fix P', like 'fix P by binding to mineral surfaces' or whatever. Some background on fixation is more warranted considering the following paragraph on mobilizing P.

We agree that complementary information would be useful to the reader. Some explanations about P fixation in acidic and calcareous soils were added to the text.

~~100~~104: Programs surely focus on yield stability, but this seems to suggest they'd focus on root architecture stability over plasticity. I really don't think there is any evidence for that. Indeed, arguably some portion of observed yield stability is actually due to phenotyping plasticity. I think it's good to discuss these issues, but be careful of claims if you can't back them up with citations.

We agree and rephrased in the text. The intention was not to claim that stability of RSA was favoured but that the focus was on yield stability, leaving plasticity aside. Indeed, it was shown that root system plasticity can be involved in yield stability. References were added.

~~146~~164: Perhaps you could define these phosphate sources earlier along with which is soluble by what mechanism. I think a sort of 'methods introduction' would be warranted to explain the overall rationale and to introduce some of the terms as a brief overview of which the section below it are details. I think this would help the reader a lot to have somewhere to refer to when they forget abbreviations and such in this section.

We agree. Information was added to the introduction and an introduction to the Material & Methods section was added.

~~157~~175: Given this is a general audience, I'd rethink terms like axenic in headings and would

define when used (or define all the terms in the introduction?). I did not know this term either. Otherwise I feel I understand the experiments just fine.

We agree and the term was removed. We realize it was the only time the term “axenic” was used in the whole manuscript. We consider it not necessary.

~~175~~212: Perhaps it would be more useful to express everything in molarity of P? In the results it says shoot mass when supplemented with other P sources TCP and HA went up, but this should not be surprising I guess since it's additional P? It's hard to tell how much additional P is added.

As TCP and HA are poorly soluble, they are not in solution but in suspension when applied to the plant and the bacteria. That is the reason why their quantity was expressed in g/l. However, in case TCP and HA are completely solubilized, the P concentration in the solution would become non-limiting for plant growth (the maximum P concentration is 6.4 mM in P-/TCP treatment and 6.0 mM in P-/HA treatment). This information was added to the text.

~~178~~218: The experimental design is not described well enough to be reproducible. Were the 6 experiments simultaneous or staggered over time? For the 5 plants per treatment, are those replicates or 5 plants in the same container? How many plants per container? What was the experimental design then, RCBD, etc? Not clear how 90 plants is arrived at. What about the different bacterial lines used - was it a factorial design with the P levels?

Yes, the 6 independent experiments were spaced over time. The 5 plants cultivated for each treatment are replicates, as specified earlier in the paragraph “One plantlet was placed into each Magenta box”. For each independent experiment, we indeed used a factorial design with 6 levels for the bacterial treatment and 3 levels for the P treatment (6 different strains x 3 P treatments x 5 replicates = 90 plants per experiment). We hope the additional information, in complement of Fig 1 are helpful.

~~182~~225: Was a transparency (backlight) unit used? Were images stored in greyscale or color? What format and if jpg, what level of compression? It would also be typical to describe how roots were washed out.

We agree that information is missing. The roots were washed by gently shaking the plants in water and pulling out sticking gravels by hand. The roots were spread in a transparent tray containing a thin layer of water and scanned using a flatbed scanner equipped with a transparent unit (backlight). Grayscale (8-bit) images were saved in jpg format. The requested details were added to the text.

~~205~~249: Is persistent homology insensitive to the disturbance of the root system after washing out? In other words, measurements like length would not be influenced (as much) by different patterns of laying the washed roots in a scanning tray - but what about the persistent homology measures?

Persistent homology is a mathematical method allowing the quantification of plant morphologies (which depends on both geometry and topology). This method is not sensitive to transformation and deformation. This means that the computed persistence barcode (one barcode for each root system) is not affected by root washing and 2D scanning. The reason for this is that we chose to use a geodesic distance function to compute the persistence barcodes. As explained in Delory et al (2018), a geodesic distance value can be assigned to any point on a root system by measuring the distance along the roots (not Euclidean distance) between that point and the root system base. Therefore, because distances are

measured along the roots, the values measured on a 3D root system will be the same as the ones measured on the same root system spread in a 2D space (e.g., before scanning). These information are now in the manuscript.

An interactive figure illustrating how persistence barcodes are computed using a geodesic distance function can be found in Delory et al (2018, Figure 3). You can access the animation <u>here</u>.

~~225~~275: This helps somewhat explaining the experimental design but is not a replacement for the earlier request for more details.

Details were added in the introductory section of Material and Methods section as well as in the dedicated places (L 219 of the track changed manuscript). Fig 1 can also be consulted as a support.

~~226~~276: with reference to 'following factors' - I believe factors should be reserved for the independent variables and that 'response variables' would be more appropriate for these continuous dependent variables.

We agree and the term factor was replaced by variable for more clarity.

~~264~~313: Sharing the data and scripts is much appreciated.

Thank you.

~~268~~317: Imagine if the reader has skipped methods and went straight to the results and read this first sentence - 4 abbreviations!

The abbreviations TCP and HA were already defined in the list of abbreviations. P and NBRIP are now added to this list.

~~270~~319: Consider rephrasing 'best performing' to be more objective 'greatest solubilization ability' or similar. It may not be clear what 'best' means in this context.

We agree and modified the text according to your suggestion.

~~271~~320: To reiterate about adding some details, I'm not sure what would be expected for the solubility of TCP and HA so when I read the results I don't know if I should be surprised or not.

This is clarified now in the new section 2.1. TCP is more easily solubilized than HA. This is also discussed in section 4.2.

~~273~~323: 'stationary populations' is not overly clear, maybe you mean 'stable population sizes'?

Yes. The text was modified according to your suggestion for more clarity.

~~287~~336: Is 'significantly' used here in a statistical sense or an effect size? Consider 'substantially' instead if warranted. Consider use of significant throughout, perhaps it is not always necessary given both the effect size and p values are given.

“Significantly” is only used in a statistical sense throughout the manuscript. We agree that it can be redundant with the p-values also present in the text. The term was removed where necessary.

~~337~~376: It's not clear what metric 'morphology' refers to. Somewhere, maybe in methods or here, you need to explain what the metric means - complexity? branchiness?

Root system morphology can be described in different ways. So far, root systems have mainly been characterised using geometric descriptors such as total root length or number of root tips for instance. However, such descriptors often fail to describe the full complexity of root systems. The main reason for this is that another component of root system morphology, which is topology (i.e., the way individual roots are connected to each other through branching), is often not taken into account. Two root systems can look very similar from a geometric perspective (same total root length and same number of root tips), but have very different topologies.

Persistent homology is a mathematical method allowing the quantification of plant morphology. The idea behind this method is to create a barcode encapsulating geometric and topological information for each root system (see interactive Figure 3 in Delory et al. 2018). For instance, the number of horizontal bars in a barcode is equal to the number of root tips, and the sum of the length of all horizontal bars in a barcode is equal to the total root length. In addition, topological information is encapsulated by the birth/death of each branch along a geodesic distance function.

We argue that an analysis of plant root systems using persistent homology nicely complements more classic approaches relying on geometric descriptors.

~~372~~421: I think this may be the only time that solubility level of the nutrients was explained. So this should be in the method intro I recommended and also explain why then it's informative to use both TCP and HA?

The solubility level of the P forms is now also explained in sections 2.1 and 2.4. More information about the choice of TCP and HA is also available in section 2.1. We hope this allows an easier reading.

~~382~~428: Clearly a reduction in root biomass would be expected as the levels are more limiting. You would have to compare absolute P content available and concentrations to discuss more.

We disagree that a reduction in root biomass would have clearly been expected. Contrasted responses are found in the literature. In addition to the articles cited in the manuscript, Liu et al. (Crop and Pasture Science, 67 (8), 2016) reported a decrease in root biomass of maize but a steady root biomass in faba bean under P-limiting condition.

The P concentration in the solution in case TCP and HA were completely solubilized was added in the material and methods section. The P concentration in the solution would have become non-limiting for plant growth (the maximum P concentration is 6.4 mM in P-/TCP treatment and 6.0 mM in P-/HA treatment).

~~384~~431: greenhouse conditions don't really have much to do with plant age if that's your point? You can grow old or young plants in any environment.

We agree that plants of any ages can be grown in different environments. However, from an allometric point of view, it is acknowledged that plants develop more roots when they are small and develop more their aboveground part when they become larger (Weiner 2004). The expression “younger growth stage” that we used refers more to the plant size than its age. By comparing the biomass of plants in the study of Poiré and in ours, it appears that the

plants were smaller in our study, potentially allocating more biomass to the roots. This complementary information was added to the manuscript.

393: suggest replacing compartment with system?

We agree (see next comment).

~~396~~444: I'm not sure why you are using compartment in this way. I think because of the allocation, but to me shoot and root system are sufficient, no need to tag on compartment or any other word.

We agree that “root system” is more commonly used. The term compartment was replaced by system or removed where we found it appropriate.

~~434~~478: Interesting discussion to me, and as a side note (no response needed), follow up studies could include adding growth substrate for bacteria to the media. Indeed, it seems natural soil would have some substrates available as opposed to this rather barren substrate (that many people commonly use similarly). Or, if exudation can be primed in other ways? Plant mutants?

Thank you for these suggestions. Indeed, a source of sugar could be added to the growth medium but caution must be paid as the plant growth and its response to P starvation will probably also be affected. A well characterized natural soil is also an interesting further step in characterizing bio-inoculants activity. Regarding the priming of exudation to feed the bacteria, the impact on the exudation response to P starvation should also be considered.

~~453~~551: Again, the persistent morphology metric is not well described. I understand it is fairly abstract, but I thought related to something like complexity so you might infer something about that?

As persistent homology encapsulates geometrical and topological information, we can infer that the morphology is impacted in one of these components at least. The TRL, being significantly reduced in plants inoculated with Pfl29Arp, is partly responsible for this modulation of morphology.
